# Supplementary material for: Integrated analysis of copy number variation-associated lncRNAs identifies candidates contributing to the etiologies of congenital kidney anomalies
Source: Commun Biol. 2023 Jul 17;6:735. doi: 10.1038/s42003-023-05101-9 (PMC10352346; doi:10.1038/s42003-023-05101-9)
Supplement: Supplementary file 2 — Supplementary Information [file 42003_2023_5101_MOESM2_ESM.pdf]

Integrated analysis of copy number variation-associated lncRNAs identifies candidates contributing to the etiologies of congenital kidney anomalies

Yibo Lu[1†], Yiyang Zhou[1†], Jing Guo[1†], Ming Qi[1], Yuwan Lin[1], Xingyu Zhang[1]
Ying Xiang[1,2*], Qihua Fu[1,2*] & Bo Wang[1,2*]

Affiliations:

[1]Pediatric Translational Medicine Institute, Shanghai Children's Medical Center, School of Medicine, Shanghai Jiao Tong University, Shanghai 200127, China

[2]Shanghai Key Laboratory of Clinical Molecular Diagnostics for Pediatrics, Shanghai 200127, China

[†] These authors contributed equally.

*Corresponding author. Email: booew@163.com; qfu@shsmu.edu.cn; 1262975038@qq.com

---

Supplementary information

Supplementary Figure 1. Recurrent CNVs associated with at least two developmental disorders (n = 18).
Supplementary Figure 2. RNA-seq analyses of *HSALNG0134318* knockdown effect on gene expression in the HEK293 cell line.
Supplementary Figure 3. *HSALNG0134318* knockdown effect on the biological processes, cellular components and molecular functions of the HEK293 cell line.
Supplementary Figure 4. Two hub CNV-lncRNAs (*HSALNG0134318* and *HSALNG0115943*) were correlated with CAKUT genes during kidney development and kidney organoids differentiation.
Supplementary Figure 5. CAKUT genes involved CNV-lncRNA-miRNA-mRNA regulatory network.
Supplementary Figure 6. miRNA interactions with CAKUT genes and CAKUT associated CNV-lncRNAs.
Supplementary Table 1. Expression patterns of known CAKUT genes (n = 172).
Supplementary Table 2. Transcription factors that potentially interacted with hub CNV-lncRNA *HSALNG0134318*.
Supplementary Table 3. CAKUT genes involved in at least two developmental disorders (n = 32).
Supplementary Table 4. The silencer sequences for transient transfection in *HSALNG0134318* and *HSALNG0115943* knockdown experiments.
Supplementary Table 5. Primers for quantitative reverse transcription qPCR analyses.

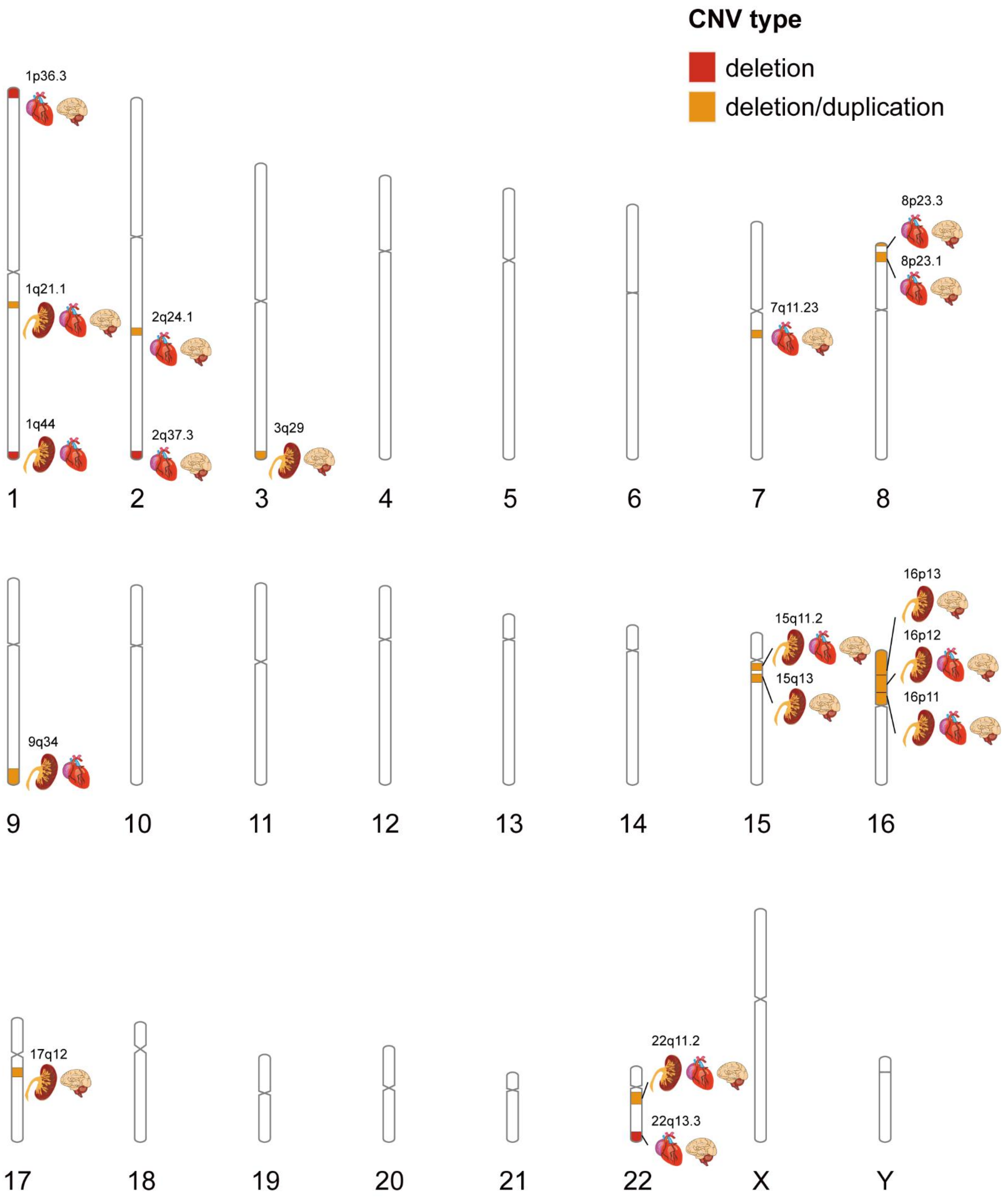

Supplementary Figure 1. Recurrent CNVs associated with at least two developmental disorders (n = 18). Map of CNVs on human genome are shown. Related developmental disorders (Renal, cardiac and neural) are labeled. Recurrent CAKUT (Table 1), CHD[1] and NDDs (including autism spectrum disorder[2] , schizophrenia[3-5] and Epilepsy[6,7]) associated CNVs were summaried from previous clinical studies. The colors of bands represent CNV type. CAKUT, congenital anomalies of the kidney and urinary tract; CHD, congenital heart disease; NDDs, neurodevelopmental disorders.

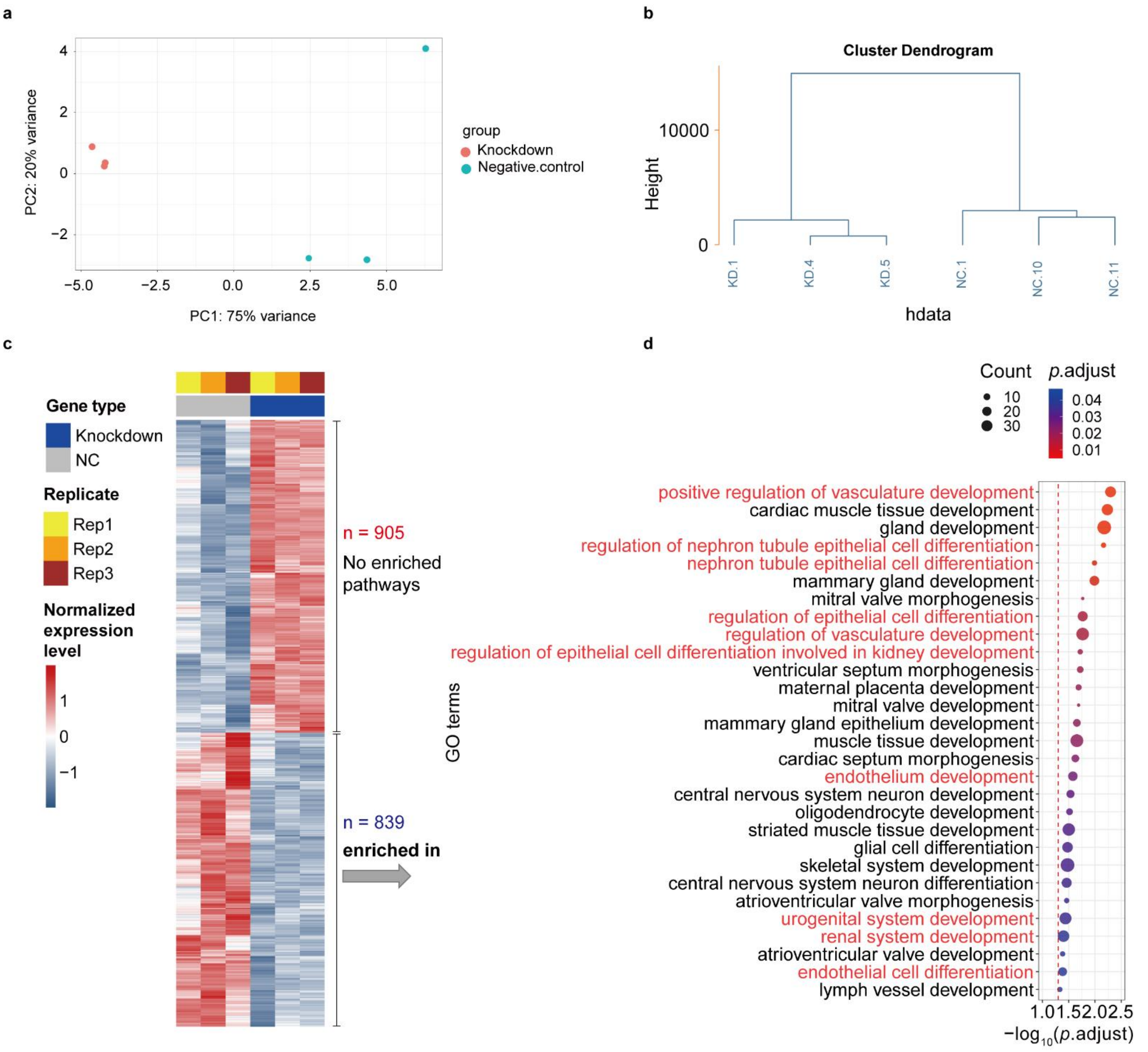

Supplementary Figure 2. RNA-seq analyses of *HSALNG0134318* knockdown effect on gene expression in the HEK293 cell line. **a and b** Principal component analysis (**a**) and hierarchical clustering (**b**) were performed to *HSALNG0134318* knockdown group ($n = 3$) compared to control group ($n = 3$) in HEK293 cell lines. The colors of dots represent different group. It reveals that *HSALNG0134318* knockdown samples were clustered well (Supplementary Data 8). **c** heatmap shows expression landscape of significantly differentially expressed genes in *HSALNG0134318* knockdown group ($n = 3$) compared to control group ($n = 3$) in the HEK293 cell lines (Supplementary Data 8). Totally 905 genes were up-regulated ($\log_2$foldchange > 0, $P_{adj} < 0.05$) and 839 genes were down-regulated ($\log_2$foldchange < 0, $P_{adj} < 0.05$). **d** Organ development-associated functional annotations of down-regulated genes in *HSALNG0134318* knockdown group are shown (Supplementary Data 8). GO terms that related to kidney development are in red font. The y axis represents GO terms, and the colors of the dots represent P_{adj} value. Values of $-\log_{10}(P_{adj})$ are shown on the x axis. The red dashed line indicates P_{adj} of 0.05.

a

BP

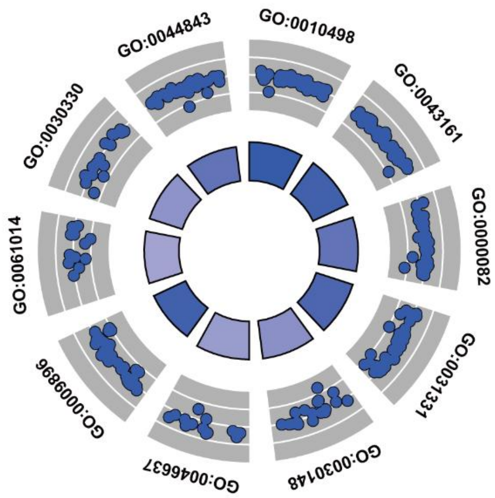

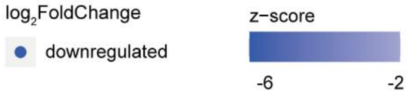

ID	Description
GO:0010498	proteasomal protein catabolic process
GO:0043161	proteasome−mediated ubiquitin−dependent protein catabolic process
GO:0000082	G1/S transition of mitotic cell cycle
GO:0031331	positive regulation of cellular catabolic process
GO:0030148	sphingolipid biosynthetic process
GO:0046637	regulation of alpha−beta T cell differentiation
GO:0009896	positive regulation of catabolic process
GO:0061014	positive regulation of mRNA catabolic process
GO:0030330	DNA damage response, signal transduction by p53 class mediator
GO:0044843	cell cycle G1/S phase transition

b

CC

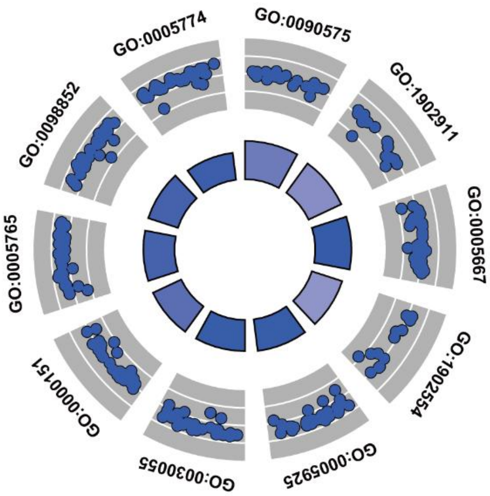

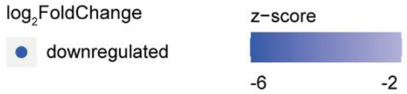

ID	Description
GO:0090575	RNA polymerase II transcription regulator complex
GO:1902911	protein kinase complex
GO:0005667	transcription regulator complex
GO:1902554	serine/threonine protein kinase complex
GO:0005925	focal adhesion
GO:0030055	cell−substrate junction
GO:0000151	ubiquitin ligase complex
GO:0005765	lysosomal membrane
GO:0098852	lytic vacuole membrane
GO:0005774	vacuolar membrane

c

MF

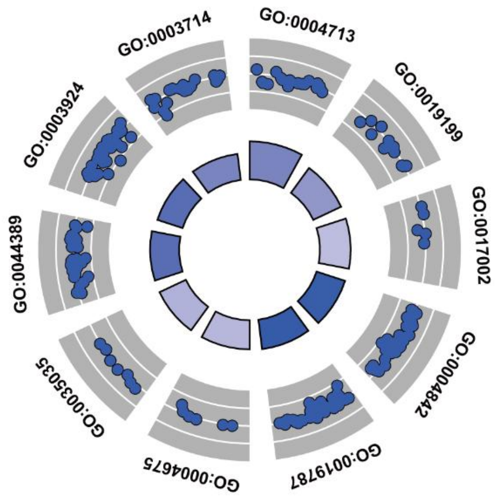

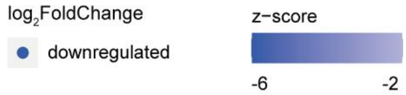

ID	Description
GO:0004713	protein tyrosine kinase activity
GO:0019199	transmembrane receptor protein kinase activity
GO:0017002	activin−activated receptor activity
GO:0004842	ubiquitin−protein transferase activity
GO:0019787	ubiquitin−like protein transferase activity
GO:0004675	transmembrane receptor protein serine/threonine kinase activity
GO:0035035	histone acetyltransferase binding
GO:0044389	ubiquitin−like protein ligase binding
GO:0003924	GTPase activity
GO:0003714	transcription corepressor activity

Supplementary Figure 3. *HSALNG0134318* knockdown effect on the biological processes, cellular components and molecular functions of the HEK293 cell line. Enriched biological process (**a**), cellular component (**b**) and molecular function (**c**) GO terms of down-regulated genes (log_2FoldChange < 0 and $P_{adj} < 0.05$, $n = 839$) after *HSALNG0134318* knockdown in the HEK293 cell line are shown in the GOcircle plots. The colors of bars in the inner circle represent z-score of each GO term. Distributions and colors of dots in the outer circle represent log_2FoldChange of genes enriched in each GO term. Descriptions of enriched GO terms are shown in the right panel (Supplementary Data 8).

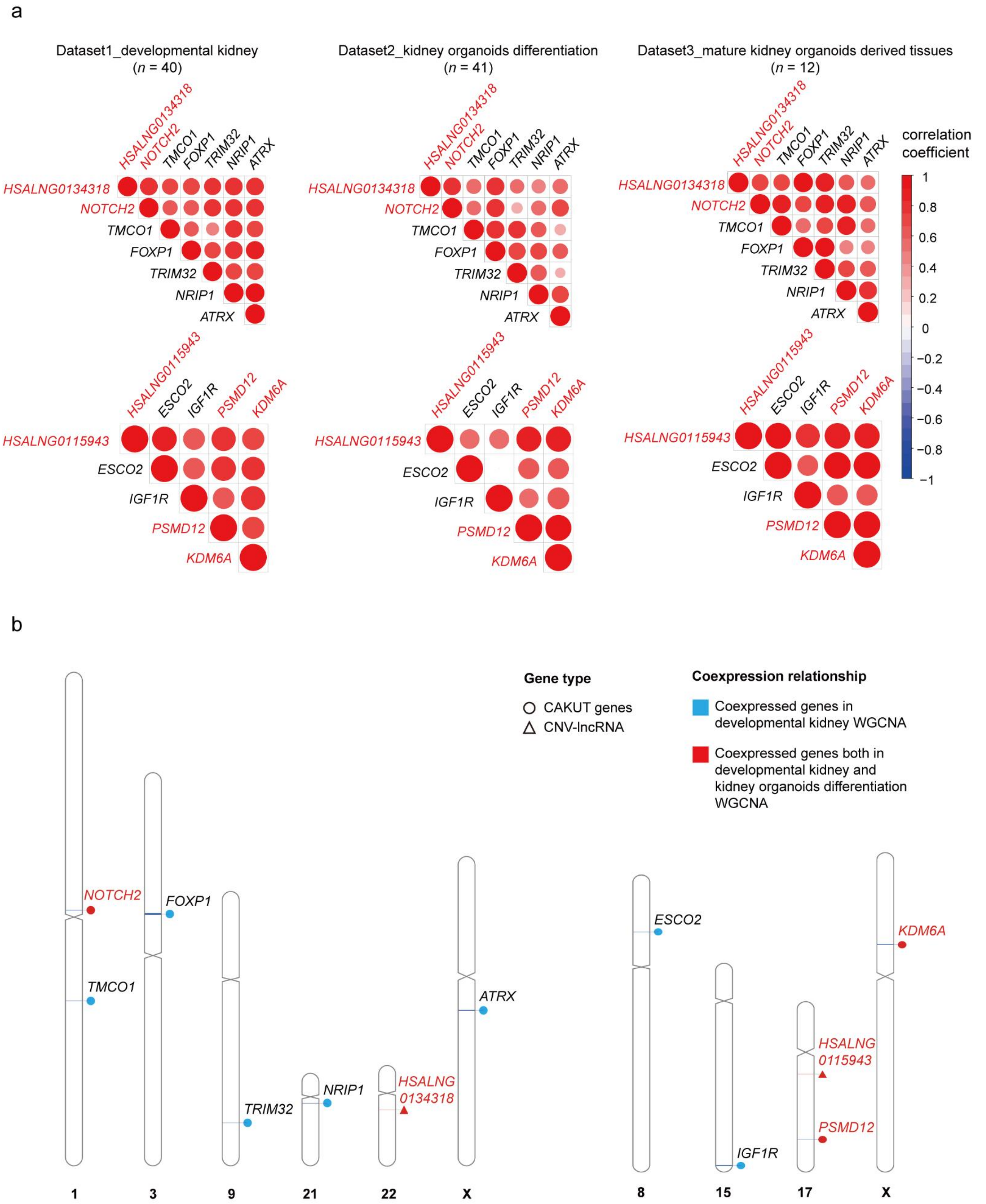

Supplementary Figure 4. Two hub CNV-lncRNAs (*HSALNG0134318* and *HSALNG0115943*) were correlated with CAKUT genes during kidney development and kidney organoids differentiation. **a** Pearson correlation coefficient between two hub CNV-lncRNAs (*HSALNG0134318* and *HSALNG0115943*) and CAKUT genes, which were significantly and positively correlated ($r \geqslant 0.5$, $P_{adj} < 0.05$) in all three datasets, are shown (Supplementary Data 11). Sizes of dots represent the absolute value of corresponding correlation coefficient. The colors of dots represent the value and direction of correlation (red: positive, blue: negative). The corresponding datasets are labeled above each panel. CAKUT genes that coexpressed with hub

CNV-lncRNAs both in kidney developmental WGCNA and kidney organoid WGCNA are highlighted in red font. **b** Distribution of two hub CNV-lncRNAs (*HSALNG0134318* and *HSALNG0115943*) and correlated CAKUT genes on human genomes are shown (Supplementary Data 11). The shapes represent gene types. The colors represent coexpression relationship type.

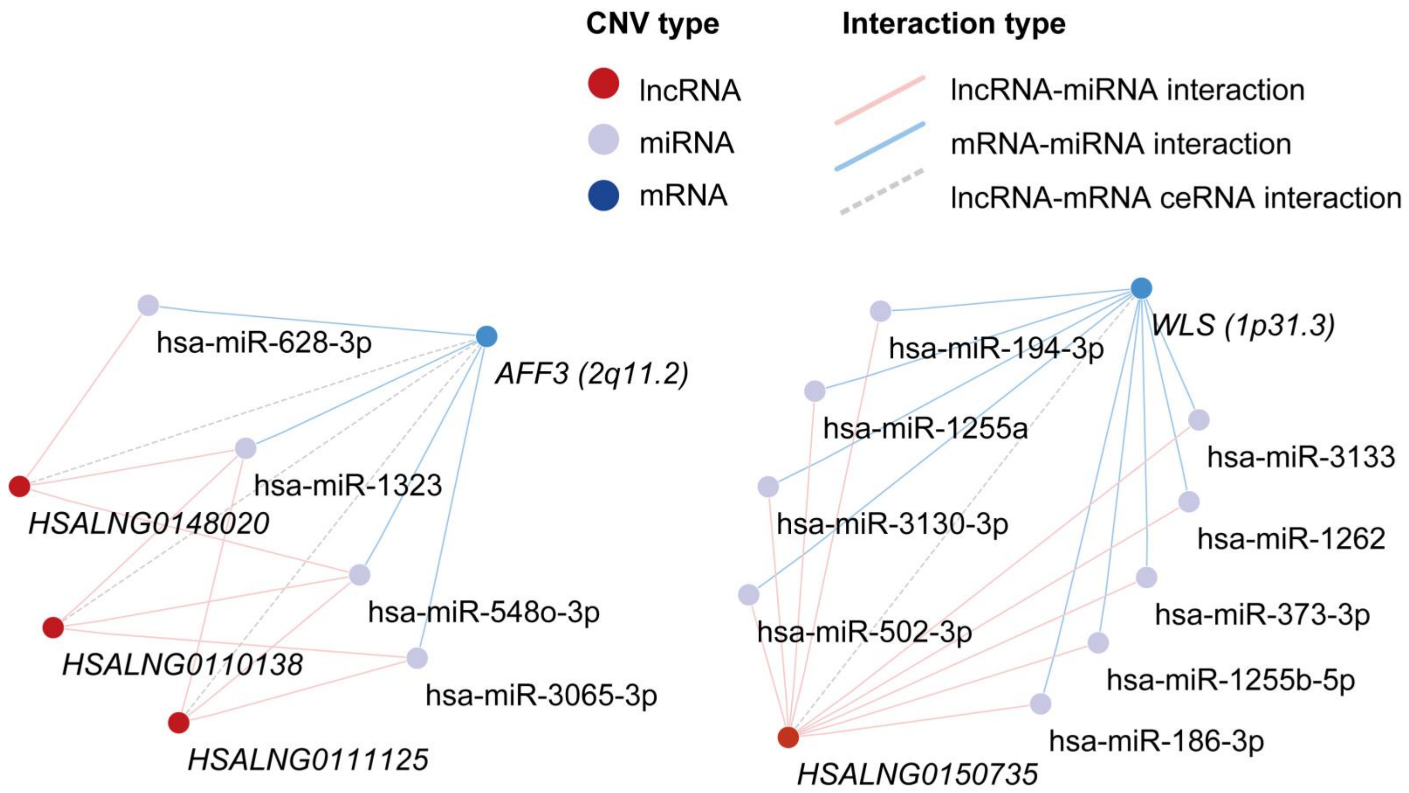

Supplementary Figure 5. CAKUT genes involved CNV-lncRNA-miRNA-mRNA regulatory network. Competing endogenous RNA (ceRNA) networks involving known CAKUT genes are shown (Supplementary Data 12). Red dots represent CNV-lncRNAs within the kidney developmental CAKUT_sig1 module. Blue dots represent mRNAs transcribed from CAKUT genes. Purple dots represent miRNAs. The red lines and blue lines represent lncRNA-miRNA and mRNA-miRNA interactions, respectively. The grey dashed lines represent ceRNA interactions.

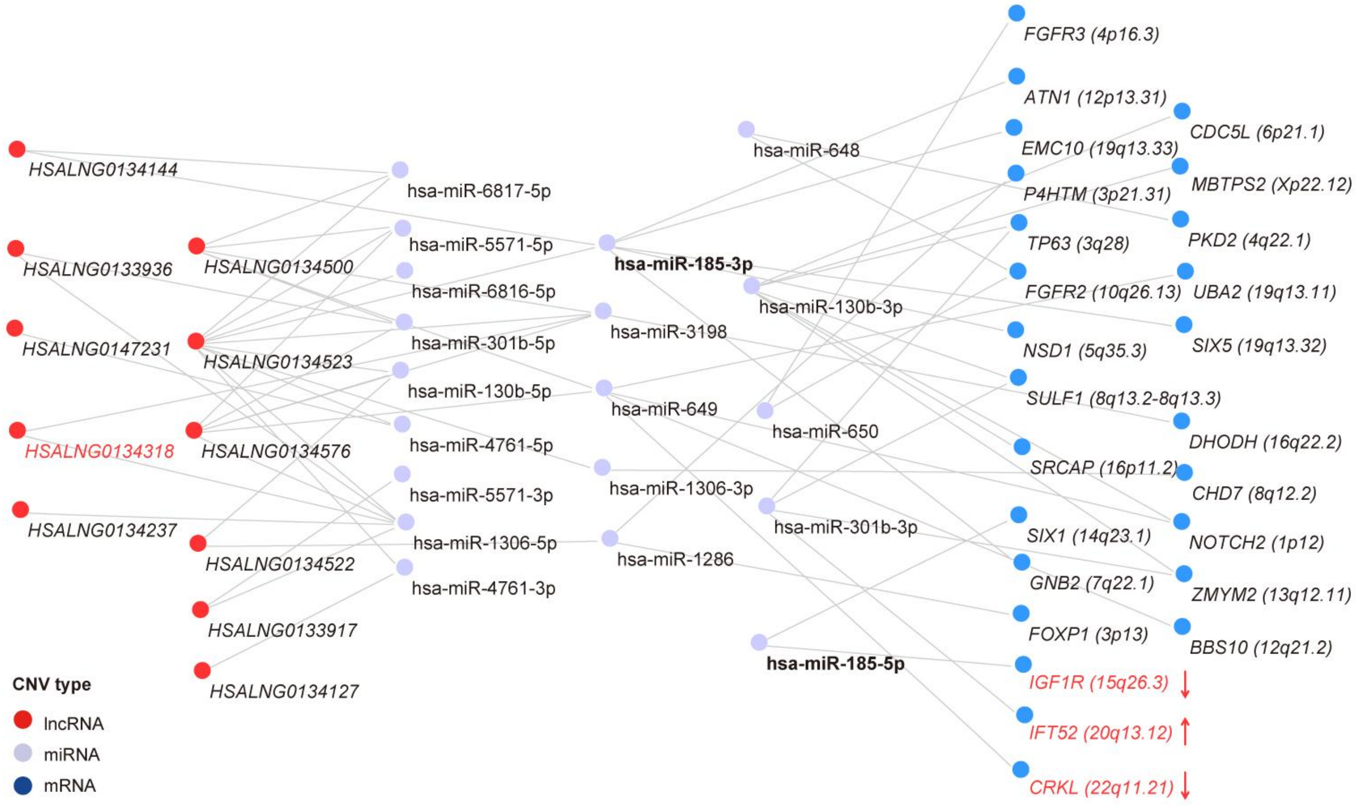

Supplementary Figure 6. miRNA interactions with CAKUT genes and CAKUT associated CNV-lncRNAs. Red dots represent CNV-lncRNAs within the kidney developmental CAKUT_sig1 module and the 22q11.2 CNV region. Blue dots represent mRNAs transcribed from CAKUT genes. For each CAKUT gene, the loci is labeled in the parenthesis. Purple dots represent miRNAs within 22q11.2 CNV region (Supplementary Data 12). hub CNV-lncRNA *HSALNG0134318* and the differential expressed CAKUT genes (*IGF1R*, *CRKL* and *IFT52*) in the *HSALNG0134318* knockdown experiments in the HEK293 cell lines are in red font (Supplementary Data 8). The up and down arrows represent up-regulated and down-regulated CAKUT genes, respectively. hsa-miR-185 (*MIR185*) that previously shown to be one of the most frequently affected miRNAs in CAKUT is in bold font[8].

Supplementary Table 1. Expression patterns of known CAKUT genes (n = 172).

Symbol	Ensembl	Expression patterns in developmental kidney (n = 40)		Expression patterns across kidney organoids differentiation (Single-end kidney organoids, day 0-25, n = 41)		CAKUT CNV	Reference
		Kidney. meanTPM	Kidney. maxTPM	kidney organoids. meanTPM	kidney. organoids. maxTPM		
ACE	ENSG00000159640	4.02	15.40	1.04	3.83		S/W/San
AFF3	ENSG00000144218	25.01	88.78	12.28	35.18		O
AGT	ENSG00000135744	17.18	121.75	20.24	95.01		S/W/San
AGTR1	ENSG00000144891	19.91	47.51	3.15	23.24		S/W/San
AGTR2	ENSG00000180772	113.94	404.50	15.41	53.61		O
ALMS1	ENSG00000116127	24.01	48.18	39.04	51.60		O
ANOS1	ENSG00000011201	12.93	38.26	46.26	231.14		H/San
ARHGAP31	ENSG00000031081	17.80	31.42	13.64	50.31		O
ARL6	ENSG00000113966	14.18	36.84	8.99	12.80		O
ARNT2	ENSG00000172379	62.23	96.88	42.46	97.69		O
ATN1	ENSG00000111676	254.46	491.25	9.79	20.76		O
ATRX	ENSG00000085224	46.60	106.38	51.69	69.81		O
B3GLCT	ENSG00000187676	50.99	279.71	46.39	86.65		O
BBS1	ENSG00000174483	3.48	6.77	1.20	3.99		O
BBS10	ENSG00000179941	53.22	163.08	52.25	86.04		O
BBS12	ENSG00000181004	17.17	43.28	22.73	51.19		O
BBS4	ENSG00000140463	40.66	67.38	46.41	56.74		O
BBS7	ENSG00000138686	22.03	48.39	38.79	67.22		O
BBS9	ENSG00000122507	10.95	20.68	26.75	50.49		O
BMP4	ENSG00000125378	70.49	177.79	69.50	191.75		H
BMP7	ENSG00000101144	105.65	199.88	135.08	368.18		H
BRAF	ENSG00000157764	16.44	37.16	12.62	20.39		O
CANT1	ENSG00000171302	32.73	59.95	17.30	28.53		O
CAPN15	ENSG00000103326	20.10	45.53	28.90	44.19	16p13	O
CCBE1	ENSG00000183287	47.56	124.26	20.85	59.12		O
CCDC28B	ENSG00000160050	17.12	32.16	20.35	29.12		O
CDC5L	ENSG00000096401	75.72	142.39	128.22	176.78		H
CDKN1C	ENSG00000129757	11.97	78.07	67.69	156.18		O
CFTR	ENSG00000001626	0.41	0.94	0.96	3.94		O
CHD7	ENSG00000171316	26.65	56.77	57.37	135.38		San
CHRM3	ENSG00000133019	5.37	10.23	2.40	5.67		San
CHRNA3	ENSG00000080644	14.58	200.14	4.05	23.03		O
CHRNG	ENSG00000196811	3.79	17.97	5.25	19.96		O
COLEC10	ENSG00000184374	1.54	7.70	0.92	2.78		O
CREBBP	ENSG00000005339	53.86	84.46	23.58	38.04	16p13	O/San
CRKL	ENSG00000099942	93.19	145.92	158.96	195.58	22q11	San
DHCR7	ENSG00000172893	44.14	124.85	128.14	209.54		San
DHODH	ENSG00000102967	10.74	18.96	9.79	12.49		O
DIS3L2	ENSG00000144535	17.48	27.13	14.91	22.25		O
DLX5	ENSG00000105880	1.52	12.53	0.18	0.69		H
DLX6	ENSG00000006377	0.13	1.09	0.14	0.73		H
DPH1	ENSG00000108963	23.10	38.53	14.02	24.01	17p13	O
DSTYK	ENSG00000133059	32.03	58.65	31.10	49.72		O/San

Symbol	Ensembl	Kidney. meanTPM	Kidney. maxTPM	kidney organoids. meanTPM	kidney. organoids. maxTPM	CAKUT CNV	Reference
DYNC2H1	ENSG00000187240	18.74	43.32	41.41	75.55		O
ELN	ENSG00000049540	84.05	680.99	1.35	3.84		O
EMC10	ENSG00000161671	49.46	82.25	38.58	71.84		O
ERCC6	ENSG00000225830	7.92	13.24	9.28	14.96		O
ESCO2	ENSG00000171320	8.84	21.97	16.65	33.77		O
EVC	ENSG00000072840	48.73	114.96	25.37	56.65	4p16	S/W
EVC2	ENSG00000173040	9.26	14.65	12.13	18.91	4p16	S/W
EYA1	ENSG00000104313	35.42	133.56	41.79	140.43		O/S/H/W/San
FAM50A	ENSG00000071859	73.69	158.63	107.57	170.93		O
FBN2	ENSG00000138829	130.28	294.97	159.57	545.76		O
FGF10	ENSG00000070193	11.02	40.79	8.47	72.71		O
FGF20	ENSG00000078579	0.63	4.23	0.31	1.81		O/San
FGFR1	ENSG00000077782	94.87	193.25	75.08	156.46		San
FGFR2	ENSG00000066468	65.23	94.11	45.63	81.52		O
FGFR3	ENSG00000068078	119.30	405.28	97.30	398.66	4p16	O
FOXC1	ENSG00000054598	191.48	358.55	88.51	321.58		H
FOXP1	ENSG00000114861	6.42	14.24	6.90	13.44		O/San
FRAS1	ENSG00000138759	81.95	169.14	64.22	267.53		S/H/W/San/
FREM1	ENSG00000164946	121.45	233.94	103.43	363.02		O/S/W/San/
FREM2	ENSG00000150893	60.46	170.63	65.72	245.26		H/San
GATA3	ENSG00000107485	145.23	344.12	5.65	36.25		H/San
GDNF	ENSG00000168621	25.59	71.17	6.48	18.60		San
GFRA1	ENSG00000151892	22.00	56.54	3.06	7.88		S/W
GLI3	ENSG00000106571	22.82	60.06	15.48	30.09		O/S/W/San/
GNB2	ENSG00000172354	239.47	488.31	239.40	352.41		O
GPC3	ENSG00000147257	1751.48	4698.89	1124.74	2238.38		San
GREB1L	ENSG00000141449	4.44	16.41	34.55	176.28		O/San
GTF2IRD1	ENSG00000006704	43.18	70.17	30.38	51.36		O
GUCY2D	ENSG00000132518	0.21	0.67	0.17	0.64	17p13	O
HNF1B	ENSG00000275410	27.70	53.19	15.25	83.51	17q12	O/S/H/W/San
HPSE2	ENSG00000172987	1.63	9.03	1.06	4.64		San
IFT172	ENSG00000138002	34.95	54.17	26.27	51.53		O
IFT52	ENSG00000101052	93.11	144.03	122.16	163.19		O
IFT74	ENSG00000096872	40.02	95.55	53.93	73.23		O
IGF1R	ENSG00000140443	63.97	117.05	49.24	82.31		O
IL6ST	ENSG00000134352	93.69	278.15	61.57	133.08		O
INPP5E	ENSG00000148384	7.12	14.90	10.18	15.89	9q34	O
INSL3	ENSG00000248099	1.54	14.23	0.34	1.46		O
IQCB1	ENSG00000173226	62.96	147.44	69.46	98.80		S/W
ITGA8	ENSG00000077943	86.42	182.06	84.79	306.05		O
JAG1	ENSG00000101384	174.17	351.04	439.04	1448.20		O/San
JAM3	ENSG00000166086	78.89	143.11	97.20	158.73		O
KAT6B	ENSG00000156650	17.28	33.66	9.95	14.63		O
KDM6A	ENSG00000147050	29.12	59.30	39.90	99.40		San
KIF14	ENSG00000118193	17.47	44.72	54.46	93.58		O
KMT2D	ENSG00000167548	35.85	67.87	11.20	16.15		O/San
LAMB2	ENSG00000172037	217.38	370.01	148.95	429.89		S/W
LMX1B	ENSG00000136944	31.81	95.11	14.66	61.13		S/W
LRIG2	ENSG00000198799	21.44	38.85	35.92	59.80		San
LRTOMT	ENSG00000284922	0.00	0.00	0.00	0.00		O
MBTPS2	ENSG00000012174	25.45	63.75	11.77	20.98		O

Symbol	Ensembl	Kidney. meanTPM	Kidney. maxTPM	kidney organoids. meanTPM	kidney. organoids. maxTPM	CAKUT CNV	Reference
MEOX1	ENSG00000005102	44.79	220.86	90.44	492.94		O
MNX1	ENSG00000130675	0.10	0.80	0.18	0.64		San
MYOD1	ENSG00000129152	0.28	6.55	0.14	0.95		O
MYOG	ENSG00000122180	4.14	86.02	0.54	2.38		H
NADSYN1	ENSG00000172890	15.45	48.52	10.49	17.56		O
NECTIN1	ENSG00000110400	40.40	84.38	37.72	66.26		O
NIPBL	ENSG00000164190	59.65	121.15	52.39	68.72		San
NOTCH2	ENSG00000134250	129.07	232.84	180.59	453.59		San
NPHP1	ENSG00000144061	7.89	15.17	4.68	11.61		S/W
NPHP4	ENSG00000131697	7.90	15.29	8.10	11.85		S/W
NPHS1	ENSG00000161270	208.48	558.02	158.57	896.40		S/W
NPHS2	ENSG00000116218	246.61	625.48	204.73	1358.46		S/W
NRIP1	ENSG00000180530	75.16	183.85	170.43	291.11		O/San
NSD1	ENSG00000165671	63.36	121.48	88.14	130.56		O
OTUD5	ENSG00000068308	83.65	123.38	78.59	98.44		O
P4HTM	ENSG00000178467	26.64	47.95	14.86	36.37		O
PAX2	ENSG00000075891	150.37	478.03	10.27	43.43		O/S/H/W/San
PBX1	ENSG00000185630	71.84	166.42	30.12	38.45		O/San
PIEZO2	ENSG00000154864	15.94	56.53	10.41	47.25		O
PIGQ	ENSG00000007541	45.47	154.48	24.01	41.20	16p13	O
PKD1	ENSG00000008710	25.22	57.76	18.40	34.88	16p13	S/W
PKD2	ENSG00000118762	102.58	201.83	69.46	101.45		S/W
PKHD1	ENSG00000170927	26.47	57.97	2.52	18.16		S/W
PLVAP	ENSG00000130300	283.47	1159.52	176.72	730.79		O
PRODH	ENSG00000100033	3.64	22.39	3.25	16.93	22q11	O
PSMD12	ENSG00000197170	39.48	86.07	79.92	149.06		O
PUF60	ENSG00000179950	34.47	70.68	183.95	285.64		O
RAI1	ENSG00000108557	36.29	78.49	21.46	39.77		O
RBM8A	ENSG00000265241	106.59	161.24	89.72	119.12	1q21	O
RECQL4	ENSG00000160957	23.41	79.36	39.80	86.94		O
REN	ENSG00000143839	534.41	7554.95	19.36	90.79		S/W/San
RET	ENSG00000165731	22.54	56.39	0.85	3.21		O/H/San
ROBO2	ENSG00000185008	45.42	106.71	22.06	87.49		O/S/H/W/San
SALL1	ENSG00000103449	159.38	340.52	162.30	620.58		O/S/H/W/San
SALL4	ENSG00000101115	18.81	39.49	165.52	400.16		O/San
SDCCAG8	ENSG00000054282	9.89	16.63	19.85	33.15	1q44	O
SEMA3A	ENSG00000075213	16.65	49.40	40.34	119.56		San
SF3B2	ENSG00000087365	117.04	196.13	152.14	173.00		O
SIX1	ENSG00000126778	12.30	45.01	32.49	88.34		O/S/H/W/San
SIX2	ENSG00000170577	100.63	393.10	5.00	16.20		H
SIX4	ENSG00000100625	13.74	48.46	39.47	77.78		S/W
SIX5	ENSG00000177045	47.77	103.90	39.59	70.30		O/S/H/W/
SLCO5A1	ENSG00000137571	2.06	5.38	3.49	10.18		O
SLIT2	ENSG00000145147	55.75	175.44	20.84	60.53		O/H/San
SLX4	ENSG00000188827	9.71	20.50	10.72	16.87	16p13	O
SMOC1	ENSG00000198732	5.94	27.89	66.67	575.40		O
SOX17	ENSG00000164736	12.07	26.95	19.66	88.32		O/San
SOX9	ENSG00000125398	51.02	130.97	9.40	34.45		S/W
SRCAP	ENSG00000080603	2.92	5.39	1.25	2.19	16p11	O
SRGAP1	ENSG00000196935	12.60	27.09	4.47	13.56		O/San
STRA6	ENSG00000137868	32.92	167.61	33.41	188.16		O
SULF1	ENSG00000137573	44.88	113.32	77.40	394.45		O

Symbol	Ensembl	Kidney. meanTPM	Kidney. maxTPM	kidney organoids. meanTPM	kidney. organoids. maxTPM	CAKUT CNV	Reference
TBC1D1	ENSG00000065882	80.04	115.11	81.04	172.80		San
TBX1	ENSG00000184058	2.66	18.60	3.49	31.75	22q11	O
TBX18	ENSG00000112837	16.10	73.42	4.12	24.70		O/San
TCTN2	ENSG00000168778	33.90	50.97	24.24	38.06	12q24	O
TCTN3	ENSG00000119977	106.03	164.49	119.90	158.52		O
TFAP2A	ENSG00000137203	24.83	51.08	0.52	2.44		O
TMCO1	ENSG00000143183	80.91	140.36	98.29	155.34		O
TMEM218	ENSG00000150433	20.63	42.22	12.90	16.43		O
TMEM260	ENSG00000070269	26.60	51.05	15.75	26.75		O
TMEM67	ENSG00000164953	14.85	32.22	16.78	30.33		O
TNPO2	ENSG00000105576	89.97	174.25	72.37	90.05		O
TP63	ENSG00000073282	1.36	8.25	0.29	0.93		H
TRAP1	ENSG00000126602	75.41	130.30	79.71	126.17	16p13	San
TRIM32	ENSG00000119401	35.00	65.14	33.01	50.33		O
UBA2	ENSG00000126261	157.43	335.29	283.09	395.08		O
UBE3B	ENSG00000151148	23.94	32.54	22.02	33.54	12q24	O
UPK3A	ENSG00000100373	5.54	67.54	0.14	0.98		O/H
USF2	ENSG00000105698	84.78	135.11	74.32	100.17		H
VANGL1	ENSG00000173218	14.65	25.17	22.00	31.33		San
WDR19	ENSG00000157796	44.57	73.93	33.76	91.38		O
WLS	ENSG00000116729	123.26	237.05	104.46	234.00		O
WNT4	ENSG00000162552	18.27	103.44	6.58	23.77		San
WNT5A	ENSG00000114251	43.15	164.91	80.22	479.92		O
WT1	ENSG00000184937	144.82	282.77	99.52	259.20		S/W
ZIC3	ENSG00000156925	0.05	1.28	26.31	196.06		O
ZMYM2	ENSG00000121741	47.87	117.49	76.38	102.13		O

Note: O, OMIM database[9](Key words: Renal anomalies; CAKUT); S, Schedl A. et al. Nat Rev Genet. 2007[10]; H, Hildebrandt, Friedhelm. Lancet. 2010[11]; W, Westland, Rik et al. Clin J Am Soc Nephrol. 2014[12]; San, Sanna-Cherchi, Simone et al. J Clin Invest. 2018[13].

Supplementary Table 2. Transcription factors that potentially interacted with hub CNV-lncRNA *HSALNG0134318*.

TF NAME [ID]	SYMBOL	ENSEMBL	is known CAKUT gene	Module (Kidney developmental)
AhR:Arnt [T05394]	*AHR*	ENSG00000106546		greenyellow
AhR:Arnt [T05394]	*ARNT*	ENSG00000143437		CAKUT_sig1
AP-2alphaA [T00035]	*TFAP2A*	ENSG00000137203	T	CAKUT_sig1
AR [T00040]	*AR*	ENSG00000169083		green
ATF-2 [T00167]	*ATF2*	ENSG00000115966		CAKUT_sig1
C/EBPalpha [T00105]	*CEBPA*	ENSG00000245848		blue
C/EBPbeta [T00581]	*CEBPB*	ENSG00000172216		yellow
c-Ets-1 [T00112]	*ETS1*	ENSG00000134954		greenyellow
c-Ets-2 [T00113]	*ETS2*	ENSG00000157557		yellow
c-Myb [T00137]	*MYB*	ENSG00000118513		CAKUT_sig1
CREB [T00163]	*CREB1*	ENSG00000118260		CAKUT_sig1
E2F [T00221]	*E2F1*	ENSG00000101412		brown
Elk-1 [T00250]	*ELK1*	ENSG00000126767		blue
ER-alpha [T00261]	*ESR1*	ENSG00000091831		black
FOXP3 [T04280]	*FOXP3*	ENSG00000049768		blue
GATA-1 [T00306]	*GATA1*	ENSG00000102145		blue
GATA-2 [T00308]	*GATA2*	ENSG00000179348		blue
GCF [T00320]	*GCFC2*	ENSG00000005436		CAKUT_sig1
GR [T05076]	*NR3C1*	ENSG00000113580		CAKUT_sig1
GR-alpha [T00337]	*NR3C1*	ENSG00000113580		CAKUT_sig1
GR-beta [T01920]	*NR3C1*	ENSG00000113580		CAKUT_sig1
HNF-1A [T00368]	*HNF1A*	ENSG00000135100		black
HNF-3alpha [T02512]	*FOXA1*	ENSG00000129514		steelblue
HOXD10 [T01425]	*HOXD10*	ENSG00000128710		CAKUT_sig1
HOXD9 [T01424]	*HOXD9*	ENSG00000128709		blue
IRF-1 [T00423]	*IRF1*	ENSG00000125347		yellow
LEF-1 [T02905]	*LEF1*	ENSG00000138795		CAKUT_sig1
NF-1 [T00539]	*NF1*	ENSG00000196712		CAKUT_sig1
NF-AT1 [T00550]	*NFATC2*	ENSG00000101096		blue
NF-AT1 [T01948]	*NFATC2*	ENSG00000101096		blue
NFI/CTF [T00094]	*NFIC*	ENSG00000141905		pink
NF-Y [T00150]	*NFYA*	ENSG00000001167		CAKUT_sig1
p53 [T00671]	*TP53*	ENSG00000141510		brown
Pax-5 [T00070]	*PAX5*	ENSG00000196092		yellow
PEA3 [T00685]	*ETV4*	ENSG00000175832		brown
PR A [T01661]	*PGR*	ENSG00000082175		midnightblue
PR B [T00696]	*PGR*	ENSG00000082175		midnightblue
RXR-alpha [T01345]	*RXRA*	ENSG00000186350		blue
Sp1 [T00759]	*SP1*	ENSG00000185591		CAKUT_sig1
SRY [T00997]	*SRY*	ENSG00000184895		grey
STAT4 [T01577]	*STAT4*	ENSG00000138378		floralwhite
T3R-beta1 [T00851]	*THRB*	ENSG00000151090		saddlebrown
TBP [T00794]	*TBP*	ENSG00000112592		CAKUT_sig1
TCF-4 [T02918]	*TCF4*	ENSG00000196628		CAKUT_sig1
TCF-4E [T02878]	*TCF4*	ENSG00000196628		CAKUT_sig1
TFIID [T00820]	*TBP*	ENSG00000112592		CAKUT_sig1
TFII-I [T00824]	*GTF2I*	ENSG00000263001		CAKUT_sig1
YY1 [T00915]	*YY1*	ENSG00000100811		CAKUT_sig1

Note: TF, transcription factor.

Supplementary Table 3. CAKUT genes involved in at least two developmental disorders (*n* = 32).

Symbol	Ensembl	Module (Kidney developmental)	CAKUT genes	CHD genes	NDDs genes	Expression patterns in developmental kidney, heart, brain and cerebellum: Kidney meanTPM	Heart meanTPM	Brain meanTPM	Cerebellum meanTPM	Loci
AFF3	ENSG00000144218	CAKUT_sig1	✔		✔	25.01	10.60	45.37	36.85	2q11.2
ATRX	ENSG00000085224	CAKUT_sig1	✔		✔	46.60	27.91	56.41	37.00	Xq21.1
B3GLCT	ENSG00000187676	purple	✔	✔		50.99	27.96	18.90	8.81	13q12.3
BRAF	ENSG00000157764	CAKUT_sig1	✔	✔	✔	16.44	8.64	18.91	20.02	7q34
CHD7	ENSG00000171316	brown	✔	✔	✔	26.65	16.77	62.44	75.20	8q12.2
CREBBP	ENSG00000005339	brown	✔	✔	✔	53.86	31.35	57.45	43.95	16p13.3
DHCR7	ENSG00000172893	blue	✔	✔	✔	44.14	27.75	64.23	38.53	11q13.4
ESCO2	ENSG00000171320	CAKUT_sig1	✔	✔		8.84	4.04	7.41	0.54	8p21.1
EVC	ENSG00000072840	darkred	✔	✔		48.73	12.13	8.96	2.88	4p16.2
EVC2	ENSG00000173040	blue	✔	✔		9.26	2.72	3.31	0.71	4p16.2
FBN2	ENSG00000138829	CAKUT_sig1	✔	✔		130.28	193.34	37.02	1.41	5q23.3
FOXC1	ENSG00000054598	CAKUT_sig1	✔	✔		191.48	28.86	24.12	10.04	6p25.3
FOXP1	ENSG00000114861	CAKUT_sig1	✔		✔	6.42	9.09	8.15	3.15	3p13
JAG1	ENSG00000101384	CAKUT_sig2	✔	✔		174.17	45.86	45.99	12.09	20p12.2
KAT6B	ENSG00000156650	CAKUT_sig1	✔	✔		17.28	9.09	18.99	10.16	10q22.2
KDM6A	ENSG00000147050	CAKUT_sig1	✔	✔		29.12	25.25	19.94	16.50	Xp11.3
KMT2D	ENSG00000167548	brown	✔	✔		35.85	22.65	33.64	39.02	12q13.12
NIPBL	ENSG00000164190	CAKUT_sig1	✔	✔	✔	59.65	34.95	54.06	32.09	5p13.2
NOTCH2	ENSG00000134250	CAKUT_sig1	✔	✔		129.07	38.66	28.76	9.69	1p12
NSD1	ENSG00000165671	CAKUT_sig1	✔	✔	✔	63.36	32.35	53.96	39.62	5q35.3
PKD1	ENSG00000008710	brown	✔	✔		25.22	28.99	65.53	240.36	16p13.3
PSMD12	ENSG00000197170	CAKUT_sig1	✔		✔	39.48	32.05	38.75	28.00	17q24.2
PUF60	ENSG00000179950	blue	✔		✔	34.47	24.50	54.23	33.72	8q24.3
RAI1	ENSG00000108557	blue	✔		✔	36.29	27.20	54.85	27.66	17p11.2
RECQL4	ENSG00000160957	brown	✔	✔		23.41	19.19	37.68	22.00	8q24.3
SALL1	ENSG00000103449	CAKUT_sig1	✔	✔		159.38	9.61	52.06	7.59	16q12.1
SRCAP	ENSG00000080603	blue	✔		✔	2.92	1.69	2.82	2.42	16p11.2
STRA6	ENSG00000137868	brown	✔	✔		32.92	7.89	7.64	1.18	15q24.1
SULF1	ENSG00000137573	magenta	✔	✔		44.88	33.28	33.47	2.86	8q13.2-8q13.3
TBX1	ENSG00000184058	magenta	✔	✔		2.66	1.59	1.97	0.63	22q11.21
TBX18	ENSG00000112837	magenta	✔	✔		16.10	29.18	4.82	2.83	6q14.3
ZIC3	ENSG00000156925	green	✔	✔		0.05	0.04	43.52	46.29	Xq26.3

Note: CAKUT, congenital anomalies of the kidney and urinary tract; CHD, congenital heart disease; NDDs, neurodevelopmental disorders. Reference of CAKUT, CHD and NDDs gene set was listed in the **METHODS** (See '**Known CAKUT, CHD and NDDs gene sets**' section; Supplementary Data 16).

Supplementary Table 4. The silencer sequences for transient transfection in *HSALNG0134318* and *HSALNG0115943* knockdown experiments.

	**The silencer sequences**
HSALNG0134318	CCACTCCTAAGATTTTTCT
	GGTTTGGAGTATATACGTA
	CCTTTAAGACCCTATCACT
	GTGGTTTGGAGTATATACGT
	CCTTTAAGACCCTATCACTC
	CTTTGTGCTTTTCCTTTCAT
HSALNG0115943	CACAATAAGAGTCATTGGCC
	AATCTGGCCCATCACGCACC
	AGCTCACCTAAACAGCCTAG
	GAGGCATAAGCTCACCTAA
	CCTGGATGCCAAATAGACA
	CGAGGAGTCCTTGCAATCT

Supplementary Table 5. Primers for quantitative reverse transcription qPCR analyses.

Gene	Primer1 (5'-3')	Primer2 (5'-3')
Lnc1 (HSALNG0134318)	TCCCCAGCTGTGAAAGGAGG	GGCCCGATTTTCCCTTGTGC
Lnc2 (HSALNG0115943)	CCCCAGGACAAGCGCAAAGT	ACCAAACTTCCGGCAAACAGT
CDC5L	GCAGCGGTAATGAAATATGGGA	CAATGATTGGAGCAATGGTCCT
CHRM3	TCACTGTTTTGCATCCTTGTTACA	CAAGGTCATTGTGACTCTCTGACAT
CRKL	AGCAATCCAGAAAAGAGTACCC	TTCACTTCGCCTTCCCAC
FOXC1	TAGCTACATCGCGCTCATCA	ACCTTGACGAAGCACTCGTT
FRAS1	AATAGCTGCCAACCAATGCTG	CAAGAGCACACACTACATGGAG
FREM2	TGAGCCAACTGTGTTTATTC	GTATAACAGACCACCATCAAC
KDM6A	TACAGGCTCAGTTGTGTAACCT	CTGCGGGAATTGGTAGGCTC
LRIG2	AAATGCAGCGGAATGGAATTAGC	CCCCTTGTTTACTCGTGTAAGGT
NIPBL	AGCAGAGACCTGATGGGCGA	TGTCGCTCTGATTCACCCCTG
NOTCH2	GGGACCCTGTCATACCCTCT	GAGCCATGCTTACGCTTTCG
NRIP1	ATTCCAACTGTGTTCCCATAGA	CCCAAGTGTTTAGCAAGGATTG
PBX1	GCTGGAGTTTGCAGAGACAC	GGTTTTTCAGTCCGGTCTCCT
PKD2	CAGAGGGGCTGCTACAGTTT	TTGAAGAGCTTAATCCAGACCA
ROBO2	[illegible]AGACCTCACAATCACCAACATTCAA[illegible]	CAGTAACGCTGTACCATCCACTGC
SEMA3A	CAGCCATGTACAACCCAGTG	ACGGTTCCAACATCTGTTCC
FOXP1	GCAGCTTTTACAGATGCAGCAGT	CTTTCCAGAGCTGCTGCAGTTC
GAPDH	GGGAAACTGTGGCGTGAT	GAGTGGGTGTCGCTGTTGA

Reference

1 Lu, Y. *et al.* Copy number variation-associated lncRNAs may contribute to the etiologies of congenital heart disease. *Commun Biol* **6**, 189, doi:10.1038/s42003-023-04565-z (2023).

2 Abrahams, B. S. *et al.* SFARI Gene 2.0: a community-driven knowledgebase for the autism spectrum disorders (ASDs). *Mol Autism* **4**, 36, doi:10.1186/2040-2392-4-36 (2013).

3 Stefansson, H. *et al.* Large recurrent microdeletions associated with schizophrenia. *Nature* **455**, 232-236, doi:10.1038/nature07229 (2008).

4 International Schizophrenia, C. Rare chromosomal deletions and duplications increase risk of schizophrenia. *Nature* **455**, 237-241, doi:10.1038/nature07239 (2008).

5 Marshall, C. R. *et al.* Contribution of copy number variants to schizophrenia from a genome-wide study of 41,321 subjects. *Nat Genet* **49**, 27-35, doi:10.1038/ng.3725 (2017).

6 Borlot, F., Regan, B. M., Bassett, A. S., Stavropoulos, D. J. & Andrade, D. M. Prevalence of Pathogenic Copy Number Variation in Adults With Pediatric-Onset Epilepsy and Intellectual Disability. *JAMA Neurol* **74**, 1301-1311, doi:10.1001/jamaneurol.2017.1775 (2017).

7 Epilepsy Phenome/Genome Project Epi, K. C. Copy number variant analysis from exome data in 349 patients with epileptic encephalopathy. *Ann Neurol* **78**, 323-328, doi:10.1002/ana.24457 (2015).

8 Mitrovic, K. *et al.* Identification and functional interpretation of miRNAs affected by rare CNVs in CAKUT. *Sci Rep* **12**, 17746, doi:10.1038/s41598-022-22749-1 (2022).

9 Hamosh, A., Scott, A. F., Amberger, J. S., Bocchini, C. A. & McKusick, V. A. Online Mendelian Inheritance in Man (OMIM), a knowledgebase of human genes and genetic disorders. *Nucleic Acids Res* **33**, D514-517, doi:10.1093/nar/gki033 (2005).

10 Schedl, A. Renal abnormalities and their developmental origin. *Nat Rev Genet* **8**, 791-802, doi:10.1038/nrg2205 (2007).

11 Hildebrandt, F. Genetic kidney diseases. *Lancet* **375**, 1287-1295, doi:10.1016/S0140-6736(10)60236-X (2010).

12 Westland, R., Schreuder, M. F., van Goudoever, J. B., Sanna-Cherchi, S. & van Wijk, J. A. Clinical implications of the solitary functioning kidney. *Clin J Am Soc Nephrol* **9**, 978-986, doi:10.2215/CJN.08900813 (2014).

13 Sanna-Cherchi, S., Westland, R., Ghiggeri, G. M. & Gharavi, A. G. Genetic basis of human congenital anomalies of the kidney and urinary tract. *J Clin Invest* **128**, 4-15, doi:10.1172/JCI95300 (2018).
