# Supplementary material for: Integrated analysis of copy number variation-associated lncRNAs identifies candidates contributing to the etiologies of congenital kidney anomalies
Source: Commun Biol. 2023 Jul 17;6:735. doi: 10.1038/s42003-023-05101-9 (PMC10352346; doi:10.1038/s42003-023-05101-9)
Supplement: Supplementary file 3 — Description of Additional Supplementary Files [file 42003_2023_5101_MOESM3_ESM.pdf]

Description of Additional Supplementary Files

File name: Supplementary Data 1 (.xlsx).
Description: The source data for figures in this article.

File name: Supplementary Data 2 (.xlsx).
Description: CAKUT associated CNV-lncRNAs and WGCNA results.

File name: Supplementary Data 3 (.xlsx).
Description: Pathway analyses of CAKUT-associated modules.

File name: Supplementary Data 4 (.xlsx).
Description: CNV-lncRNAs and co-expressed protein coding genes in the CAKUT_sig1 module.

File name: Supplementary Data 5 (.xlsx).
Description: CNV-lncRNAs and co-expressed protein coding genes in the CAKUT_sig2 module.

File name: Supplementary Data 6 (.xlsx).
Description: Expression patterns of two hub CNV-lncRNAs (HSALNG0134318 and HSALNG0115943) and CAKUT genes during kidney development.

File name: Supplementary Data 7 (.xlsx).
Description: Data of RT-qPCR analyses.

File name: Supplementary Data 8 (.xlsx).
Description: Data of RNA-seq analyses.

File name: Supplementary Data 9 (.xlsx).
Description: Results generated from single-end kidney organoids differentiation RNAseq data.

File name: Supplementary Data 10 (.xlsx).
Description: Results generated from paired-end kidney organoids differentiation RNA-seq data.

File name: Supplementary Data 11 (.xlsx).
Description: Correlation relationships between two hub CNV-lncRNAs and CAKUT genes.

File name: Supplementary Data 12 (.xlsx).
Description: : Potential gene synergy involving CAKUT associated CNV-lncRNAs.

File name: Supplementary Data 13 (.xlsx).
Description: Differential expression analyses results between kidney and heart.

File name: Supplementary Data 14 (.xlsx).
Description: Differential expression analyses results between kidney and brain.

File name: Supplementary Data 15 (.xlsx).
Description: Differential expression analyses results between kidney and cerebellum.

File name: Supplementary Data 16 (.xlsx).
Description: Genes involved in multi-organ developmental disorders.
