# Supplementary material for: Integrated analysis of copy number variation-associated lncRNAs identifies candidates contributing to the etiologies of congenital kidney anomalies
Source: Commun Biol. 2023 Jul 17;6:735. doi: 10.1038/s42003-023-05101-9 (PMC10352346; doi:10.1038/s42003-023-05101-9)
Supplement: Supplementary file 5 — Reporting Summary [file 42003_2023_5101_MOESM5_ESM.pdf]

nature portfolio

Corresponding author(s): Bo Wang

Last updated by author(s): Jul 5, 2023

Reporting Summary

Nature Portfolio wishes to improve the reproducibility of the work that we publish. This form provides structure for consistency and transparency in reporting. For further information on Nature Portfolio policies, see our Editorial Policies and the Editorial Policy Checklist.

Statistics

For all statistical analyses, confirm that the following items are present in the figure legend, table legend, main text, or Methods section.

n/a	Confirmed	
☐	☒	The exact sample size (*n*) for each experimental group/condition, given as a discrete number and unit of measurement
☐	☒	A statement on whether measurements were taken from distinct samples or whether the same sample was measured repeatedly
☐	☒	The statistical test(s) used AND whether they are one- or two-sided<br>*Only common tests should be described solely by name; describe more complex techniques in the Methods section.*
☒	☐	A description of all covariates tested
☐	☒	A description of any assumptions or corrections, such as tests of normality and adjustment for multiple comparisons
☐	☒	A full description of the statistical parameters including central tendency (e.g. means) or other basic estimates (e.g. regression coefficient) AND variation (e.g. standard deviation) or associated estimates of uncertainty (e.g. confidence intervals)
☐	☒	For null hypothesis testing, the test statistic (e.g. *F*, *t*, *r*) with confidence intervals, effect sizes, degrees of freedom and *P* value noted<br>*Give P values as exact values whenever suitable.*
☒	☐	For Bayesian analysis, information on the choice of priors and Markov chain Monte Carlo settings
☐	☒	For hierarchical and complex designs, identification of the appropriate level for tests and full reporting of outcomes
☐	☒	Estimates of effect sizes (e.g. Cohen's *d*, Pearson's *r*), indicating how they were calculated

Our web collection on statistics for biologists contains articles on many of the points above.

Software and code

Policy information about availability of computer code

Data collection	No software was used.
Data analysis	The R package WGCNA v1.70 was used for coexpression network construction and module identification. The R package clusterProfiler v3.10 was used for Gene ontology (GO) enrichment analysis and Kyoto Encyclopedia of Genes and Genomes (KEGG) pathway enrichment analysis. The R package DESeq2 v1.32 was used for differential expression analysis. GOplot v1.0.2 was used to calculate Z-score of each enriched GO term. PROMO was used for transcription factors prediction. LncmiRSRN v3.0 was used for lncRNA-miRNA-mRNA regulatory network analysis. BEDTools v2.29.2 was used for CNV-lncRNAs retrieval. For RNA-seq analysis, the raw data was processed with following tools: quality analysis and base quality filtering with FastQC v0.11.9 and Trim Galore v0.6.6, rRNA removing with SortMeRNA v4.3.4, alignment with STAR v2.7.10, reads counting with featureCounts implemented in the Subread package v2.0.3. All the tools are cited in the paper.

For manuscripts utilizing custom algorithms or software that are central to the research but not yet described in published literature, software must be made available to editors and reviewers. We strongly encourage code deposition in a community repository (e.g. GitHub). See the Nature Portfolio guidelines for submitting code & software for further information.

Data

Policy information about availability of data

All manuscripts must include a data availability statement. This statement should provide the following information, where applicable:

- Accession codes, unique identifiers, or web links for publicly available datasets
- A description of any restrictions on data availability
- For clinical datasets or third party data, please ensure that the statement adheres to our policy

The RNA-seq raw data for knockdown experiments on HSALNG0134318 in the human embryonic kidney (HEK293) cell lines that generated in this study are available in GEO: GSE223312. The source data for figures are available in Supplementary Data 1. Data generated during this study are available in Figshare (https://doi.org/10.6084/m9.figshare.23624658.v2, Supplementary Data 2-16 files). Accession code of other raw data that support the results in this work are all available in Methods section.

Research involving human participants, their data, or biological material

Policy information about studies with human participants or human data. See also policy information about sex, gender (identity/presentation), and sexual orientation and race, ethnicity and racism.

Reporting on sex and gender	No human participant was involved in this study.
Reporting on race, ethnicity, or other socially relevant groupings	n/a
Population characteristics	n/a
Recruitment	n/a
Ethics oversight	n/a

Note that full information on the approval of the study protocol must also be provided in the manuscript.

Field-specific reporting

Please select the one below that is the best fit for your research. If you are not sure, read the appropriate sections before making your selection.

☒ Life sciences ☐ Behavioural & social sciences ☐ Ecological, evolutionary & environmental sciences

For a reference copy of the document with all sections, see nature.com/documents/nr-reporting-summary-flat.pdf

Life sciences study design

All studies must disclose on these points even when the disclosure is negative.

Sample size	Sample size was deemed to be appropriate based on the tutorials of R package WGCNA. The coexpression relationships calculated in this study with larger sample sizes were deemed to be robust. Sample size of all datasets were labeled in the paper.
Data exclusions	Pre-established QC criteria were applied to the data (e.g. gene expression value in the kidney organoids samples).
Replication	Findings on coexpression relationships between CNV-lncRNAs and CAKUT genes were replicated in the human kidney developmental dataset (n = 40), kidney organoids differentiation dataset (n = 53) and in vitro experiments in the human embryonic kidney (HEK293) cell lines. For knockdown experiments on two hub CNV-lncRNAs HSALNG0134318 and HSALNG0115943 in the HEK293 cell lines, at least three biologically independent experiments were conducted for each group.
Randomization	n/a
Blinding	n/a

Reporting for specific materials, systems and methods

We require information from authors about some types of materials, experimental systems and methods used in many studies. Here, indicate whether each material, system or method listed is relevant to your study. If you are not sure if a list item applies to your research, read the appropriate section before selecting a response.

Materials & experimental systems

n/a	Involved in the study
☒	☐ Antibodies
☐	☒ Eukaryotic cell lines
☒	☐ Palaeontology and archaeology
☒	☐ Animals and other organisms
☒	☐ Clinical data
☒	☐ Dual use research of concern
☒	☐ Plants

Methods

n/a	Involved in the study
☒	☐ ChIP-seq
☒	☐ Flow cytometry
☒	☐ MRI-based neuroimaging

Eukaryotic cell lines

Policy information about cell lines and Sex and Gender in Research

Cell line source(s)	HEK293 (RRID: CVCL_0045, Cyagen, Suzhou, China).
Authentication	Morphology or genotyping.
Mycoplasma contamination	Cells tested negative for mycoplasma contamination by the commcercial vendor. Cells were not tested after receipt.
Commonly misidentified lines (See ICLAC register)	No commonly misidentified lines were used.
